# PALS-14 promotes resistance to *Nematocida parisii* infection in *Caenorhabditis elegans*

**DOI:** 10.17912/micropub.biology.001325

**Published:** 2024-10-15

**Authors:** Deevya Raman, Nicole Wernet, Spencer Gang, Emily Troemel

**Affiliations:** 1 Cell and Developmental Biology, University of California, San Diego, San Diego, CA, United States; 2 Molecular Biology Department, Colorado College, Colorado Springs, Colorado, United States

## Abstract

Microsporidia are common natural pathogens of the nematode *Caenorhabditis elegans* . Infection of *C. elegans* by the microsporidian species *Nematocida parisii* leads to induction of the Intracellular Pathogen Response (IPR), including transcriptional upregulation of 26 *pals* genes. The divergent ‘ *pals* ' sequence signature is conserved with humans, but PALS proteins have unknown biochemical functions. So far, none of the 26 induced *pals* genes have a demonstrated role in immunity. Here, we use RNAseq data, RNA interference, and CRISPR/Cas9 mutant analysis to identify the *N. parisii* -induced *pals-14* gene as an immune gene that provides defense against microsporidia infection in *C. elegans* .

**Figure 1.**
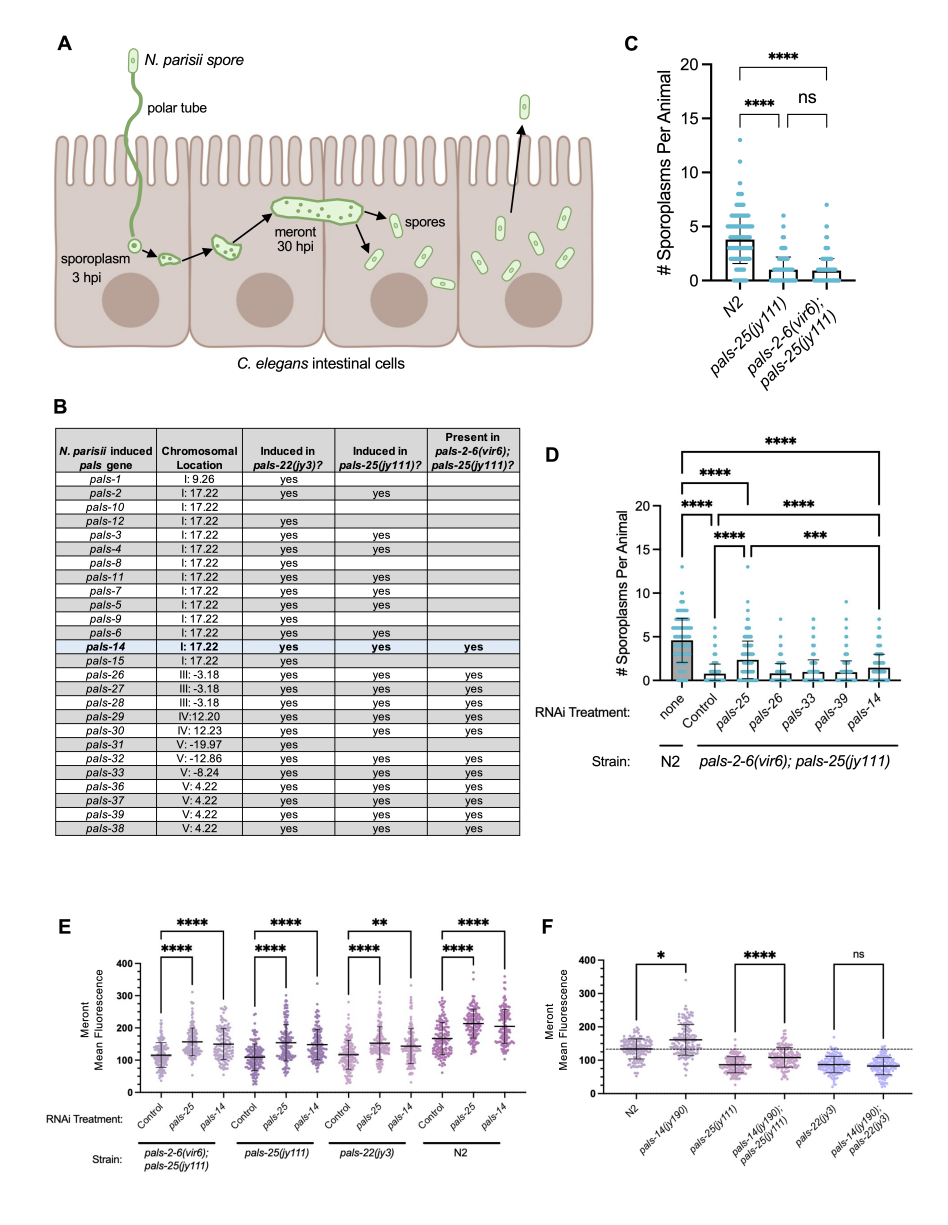
Loss of *pals-14* causes increased susceptibility to *N. parisii* infection. **A)** The lifecycle of *N. parisii* starts with an extracellular infectious spore that fires an invasion apparatus called a polar tube that delivers a parasite cell, or sporoplasm, directly into the *C. elegans* intestinal cytoplasm. This sporoplasm replicates into a multinucleated cell called a meront, which eventually differentiates back into spores that egress from the cell, exit the organism, and go on to infect new hosts. **B)** *pals* genes induced by *N. parisii* infection listed in order found in the genome, together with information about whether they are induced in *pals-22 ( jy3 )* and *pals-25 ( jy111 )* strains, based on RNAseq analysis (Gang et al., 2022). Induced *pals* genes are listed as present in *pals-2-6(vir6); pals-25 ( jy111 )* mutants if they are not deleted by the *pals-2-6(vir6)* deletion. (RNAseq analysis has not yet been performed on *pals-2-6(vir6); pals-25 ( jy111 )* mutants.) **C)** *N. parisii* sporoplasms per animal infected at the L1 stage, quantified 3 hours post-inoculation (hpi). **D)** *N. parisii* sporoplasms per animal infected at the L1 stage after RNAi treatment of the previous generation (see Methods), quantified at 3 hpi. **E)** *N. parisii* meront (pathogen) load per animal infected at the L4 stage after RNAi treatment, quantified at 30 hpi. **F)** *N. parisii* meront (pathogen) load per animal infected at the L4 stage, quantified at 30 hpi. **C-F)** Three experimental replicates were infected and quantified, with 100 animals (C&D) or 50 animals (E&F) per strain per replicate. **** *p* < 0.0001, *** *p* < 0.001, ** *p* < 0.01, * *p* < 0.05, Kruskal-Wallis test with Dunn's multiple comparisons test using Prism v10. Bars represent mean values, and error bars standard deviation. Select statistically significant comparisons are being shown for clarity.

## Description

Microsporidia are eukaryotic, single-celled, obligate intracellular pathogens that infect a broad range of hosts, including humans and agriculturally relevant hosts like honeybees, locusts and shrimp (Vavra & Lukes, 2013). Little is known about host defenses against microsporidia. The nematode *C. elegans* provides a convenient whole-animal host for studying microsporidia and probing host defense mechanisms (Gang & Lazetic, 2024; Tecle & Troemel, 2022) . *C. elegans* is naturally infected by many species of microsporidia, the most common being *Nematocida parisii* , which causes a lethal intestinal infection (Troemel et al., 2008) . The life cycle of *N. parisii* in the *C. elegans* intestine is detailed in Figure 1A .

*N. parisii* infection in *C. elegans* causes rapid transcriptional induction of hundreds of host genes (Bakowski et al., 2014; Chen et al., 2017) . This response has been termed the Intracellular Pathogen Response (IPR), and it appears to promote resistance to *N. parisii* infection (Reddy et al., 2017; Reddy et al., 2019; Tecle & Troemel, 2022) . However, which IPR-upregulated genes are important for resistance against *N. parisii* has been unclear. One subset of *N. parisii* -upregulated genes of interest is the *pals* genes, which share a *‘pals'* sequence motif of unknown function. 39 *pals* genes are in the *C. elegans* genome, compared to just one *pals* ortholog each in mice and humans (Leyva-Diaz et al., 2017). 26 *C. elegans* *pals* genes are induced by *N. parisii* infection ( Figure 1B ).

Among the 13 *pals* genes that are not induced by infection, five ( *pals-22 , pals-25 , pals-16 , pals-17 , pals-20* ) have been shown to control expression of the 26 induced *pals* genes as well as other IPR genes, acting in modules that contain positive and negative regulators (Lazetic et al., 2023; Reddy et al., 2019). One of these modules comprises the negative regulator *pals* - *22* and the positive regulator *pals-25 .* PALS-22 protein binds to and inhibits the PALS-25 protein, which activates downstream *pals* gene expression and pathogen resistance. Therefore, *pals-22* loss-of-function mutants (allele *jy3 )* and *pals-25* gain-of-function mutants (allele *jy111* ) have constitutively upregulated IPR and increased resistance to *N. parisii* . *pals-25 ( jy111 )* mutants have a C-terminal deletion of the last 13 amino acids of the PALS-25 protein (Q293*), leading to a loss of association between PALS-25 and PALS-22 (Gang et al., 2022) . This PALS-25 (Q293*) gain-of-function mutant only induces 19 *pals* genes (a subset of the 26 induced *pals* genes), but these mutants are still resistant to *N. parisii* ( Figure 1B ) (Gang et al., 2022) .

Here, we crossed *pals-25 ( jy111 )* mutants into *pals-2-6(vir6)* mutants to narrow down which induced *pals* gene(s) might contribute to pathogen resistance. *pals-2-6(vir6)* (referred to as "C17H1 deletion" in Reddy et al., 2017, with CRISPR and genotyping information in that publication) has a deletion of 11 *pals* genes on Chromosome 1, including 7 *pals* genes induced in *pals-25 ( jy111 )* mutants ( Figure 1B ) (Gang et al., 2022) . Notably, *pals-5* , one of the IPR genes highly induced and often used as a read-out for infection, is deleted in *pals-2-6(vir6)* . We then tested whether *pals-2-6(vir6); pals-25 ( jy111 )* double mutants have increased susceptibility to infection compared to *pals-25 ( jy111 )* animals. We inoculated first larval stage (L1) animals with *N. parisii* spores and then quantified the number of microsporidia sporoplasms per animal 3 hours later. Using this assay, we did not see an effect of the *pals-2-6(vir6)* deletion on susceptibility to infection, suggesting that none of the genes deleted in that strain, including *pals-5* , are required for the increased resistance observed in *pals-25 ( jy111 )* animals compared to wild-type animals ( Figure 1C ).

Given that there are 12 induced *pals* genes still present in *pals-2-6(vir6); pals-25 ( jy111 )* double mutants ( Figure 1B ), we used this strain to investigate if any of them contributes to the increased pathogen resistance of this strain. Many *pals* genes are found in clusters in the genome, so we selected 4 candidates from different genomic regions to test using RNAi-mediated knock-down followed by infection. Specifically, we performed RNAi against *pals-14* , *pals-26* , *pals-33* , and *pals-39* . Using the sporoplasm assay described above, we also included *pals-25* RNAi as a positive control, which increased susceptibility to infection, consistent with prior results. Interestingly, *pals-14* RNAi caused a modest but significant increase in sporoplasm numbers compared to the empty vector control strain ( Figure 1D ). These findings identify the previously uncharacterized *pals-14* as a candidate resistance gene against *N. parisii* .

To explore if *pals-14* has a role in resistance during other life stages of *C. elegans* and later in the developmental cycle of *N. parisii* , we inoculated fourth larval stage (L4) RNAi-treated animals with *N. parisii* spores, and 30 hours later we quantified meronts. We again found that *pals-14* RNAi caused increased susceptibility to infection in *pals-2-6(vir6); pals-25 ( jy111 )* double mutants ( Figure 1E ). We were curious if the effects of *pals-14* RNAi on pathogen load required the absence of the other *pals* genes removed by the *pals-2-6(vir6)* deletion, leading us to perform *pals-14* RNAi on *pals-25 ( jy111 )* mutants. Here, we found that *pals-14* RNAi caused increased pathogen load upon infection with *N. parisii* . Similarly, we found that *pals-14* RNAi caused increased susceptibility in *pals-22 ( jy3 )* mutants ( Figure 1E ). Furthermore, *pals-14* RNAi caused increased susceptibility in wild-type animals, indicating that it has an effect even when IPR genes are not induced before infection. Altogether, these findings indicate that *pals-14* has a role in resistance to infection that is not redundant with other *pals* genes and does not require its induction prior to infection.

To confirm the role of *pals-14* in pathogen resistance, we generated a complete deletion (allele *jy190* ) using CRISPR/Cas9-mediated editing (Dickinson & Goldstein, 2016) . We then tested the effect of *pals-14 ( jy190 )* on pathogen resistance in a wild-type background, and in *pals-25 ( jy111 )* and *pals-22 ( jy3 )* mutant backgrounds, using the L4 assay quantifying meronts. Similar to *pals-14* RNAi, we found that loss of *pals-14* in wild-type and *pals-25 ( jy111 )* mutant backgrounds led to increased pathogen load compared to wild type ( Figure 1F ). This effect was not observed in the *pals-22 ( jy3 )* mutant background, perhaps due to more IPR genes being upregulated in this strain compared to mutants in the *pals-25 ( jy111 )* background (Gang et al., 2022) . These findings confirm that *pals-14* promotes pathogen resistance against *N. parisii* in *C. elegans* in multiple strain backgrounds.

Co-evolutionary host-pathogen battles often lead to the expansion of gene families both on the host and the pathogen side (Lazetic & Troemel, 2020). The *pals* gene family expanded in *C. elegans* to include 39 members, suggesting that it might play a role in resistance to some natural pathogen(s) of *C. elegans .* Our prior work demonstrated that uninduced *pals* genes like *pals-22* and *pals-25* may play an indirect role in resistance to infection, acting through transcriptional up-regulation of hundreds of downstream genes, including induced *pals* genes like *pals-14* . Prior to this study, no induced *pals* genes had been shown to play a role in defense. Through analysis of a subset of upregulated *pals* genes combined with mutational analysis, we identified *pals-14* as the first *pals* gene involved in defense against *N. parisii* . Future studies will determine the mechanism by which *pals-14* promotes resistance against *N. parisii* in *C. elegans* .

## Methods

***C. elegans* maintenance**

*C. elegans* were maintained at 20°C on Nematode Growth Media (NGM) agar plates seeded with streptomycin-resistant *Escherichia coli* OP50-1 .

**RNA interference**

RNAi clones in the *E. coli* HT115 background were obtained from the Ahringer and Vidal libraries and sequence-confirmed before use. RNAi clones were grown overnight in LB at 37°C for Figure 1D, and at 30°C for Figure 1E, before being seeded to RNAi plates (NGM plates supplemented with 5 mM IPTG and 1 mM carbenicillin), which were grown in the dark at room temperature for 3 days before animals were added.

***N. parisii* infection**

For sporoplasm assays ( Figure 1C, 1D), adult animals were added to RNAi plates and allowed to have progeny, which were then bleached to obtain synchronized L1's. 1200 of these synchronized L1 animals were mixed with 2 million spores, OP50-1 , and M9, plated onto 6-cm NGM plates, dried, and then transferred to a 25°C incubator for 3 hours before fixation with 100% acetone.

For meront assays ( Figure 1E, 1F), synchronized L1's were added to RNAi plates and grown until the L4 stage. These worms were washed off the RNAi plates mixed with OP50-1 , M9, and 2 million *N. parisii* spores, plated on NGM plates, and immediately transferred to a 25°C incubator for a 3-hour pulse infection. After the pulse infection, the worms were washed, transferred to fresh 6-cm NGM plates with OP50-1 , and incubated at 20°C. After 27 hours, the worms were fixed in a final concentration of 4% paraformaldehyde.

Fixed worms were stained with MicroB, a Cal Fluor 610 fluorescence in-situ hybridization (FISH) probe for *N. parisii* rRNA by incubation at 46°C for 16-18 hours. For *N. parisii* assays in Figure 1C and 1D, sporoplasms were counted manually on a Zeiss AxioImager M1. For *N. parisii* assays in Figure 1E and 1F, meronts were imaged in a 96-well plate reader, and fluorescence was then quantified with ImageJ 1.54g.

**CRISPR/Cas9 generation of *pals-14 ( jy190 )* deletion mutant**

To generate *pals-14 ( jy190 )* , two CRISPR RNAs (crRNAs) were designed using Benchling, ChopChop, and IDT to remove the entire *pals-14* coding sequence (see Reagents). An injection mix containing the two crRNAs, tracrRNA, and IDT Cas9, as well as the co-injection marker *myo-2p::mCherry* , and pBluescript was injected into wild-type animals *.* *pals-14 ( jy190 )* was backcrossed three times in wildtype, *pals-22 ( jy3 )* and *pals-25 ( jy111 )* backgrounds.

***C. elegans* strains**

**Table:** 

**Strain Name**	**Genotype**	**Description**	**Source**
N2	Wild-type	Wild-type	Troemel lab collection
ERT1098	* pals-2-6(vir6)I; pals-25 ( jy111 )III *	Deletion of 11 up-regulated IPR pals genes on Chromosome 1 in a constitutive IPR activation background	Crossed *pals-2-6(vir6)I* , made by Dave Wang's lab and described as "C17H1 deletion" in *Reddy et al 2017* , with *pals-25 ( jy111 )III f* rom Gang et al., 2022; See *Reddy et al 2017* for *pals-2-6(vir6)* /C17H16 CRISPR and deletion genotyping information
ERT1316	*pals-14 ( jy190 )I *	Full deletion of *pals-14*	This study
ERT751	*pals-25 ( jy111 )III *	C-terminal truncation of *pals-25* that causes constitutive IPR activation	*Gang et al 2022*
ERT1317	*pals-14 ( jy190 )I; pals-25 ( jy111 )III *	Full-deletion of *pals-14* in *pals-25 ( jy111 )* background	This study
ERT415	*pals-22 ( jy3 )III *	G to A mutation of splice acceptor before exon 4 of *pals-22 ,* causing constitutive IPR activation	*Reddy et al 2017*
ERT1318	*pals-14 ( jy190 )I; pals-22 ( jy3 )III *	Full-deletion of *pals-14* in *pals-22 ( jy3 )* background	This study

***pals-14* CRISPR**

**Table:** 

Components	Sequence	Relative Location
CRISPR RNAs (crRNAs)	TAGATTCGTTATCTTCATAC	162 bp before 1st exon of pals-14
	CCGTGGAAATCAACATTGTC	219 bp after last exon of pals-14
pals-14 ( jy190 ) sequence	AATATGATGAAATTTAACACCCATTCCTTAG(snip)ATCAACATTGTCCAATGACTATCTGATAT	
pals-14 CRISPR Genotyping External Forward	TCAGCACTGGTAGTACCATTCC	-
pals-14 CRISPR Genotyping Internal Forward	AGTGTGTTCAACGTTGCCAC	-
pals-14 CRISPR Genotyping External Reverse	CAAAAACTTCTACATATGACCGCCA	-
pals-14 CRISPR Genotyping Internal Reverse	GTGCGATACGTTCCACATGTT	-
